# Qualitative Experiences and Depression/Anxiety Scores in Parents of Children With Cystic Fibrosis Transmembrane Conductance Regulator Related Metabolic Syndrome

**DOI:** 10.1002/ppul.71224

**Published:** 2025-08-22

**Authors:** Lynne Carty, Rebecca Dobra, Jackie Francis, Michele Puckey, Andy Bush, Jane C. Davies

**Affiliations:** ^1^ National Heart and Lung Institute Imperial College London London England UK; ^2^ Department of Paediatrics Royal Brompton Hospital, part of Guy's and St Thomas' NHS Foundation Trust London England UK

**Keywords:** CRMS/CFSPID, Cystic fibrosis, newborn screening, quality of life

## Abstract

**Background:**

Cystic Fibrosis Transmembrane Conductance Regulator Related Metabolic Syndrome/Cystic Fibrosis Screen Positive, Inconclusive Diagnosis (CRMS/CFSPID) describes children with a positive newborn screen for whom follow‐up tests neither confirm, nor definitively rule‐out, a CF diagnosis. Many are healthy carriers, but some will reclassify to a CF diagnosis; the natural history is not yet well understood. In children with chronic illnesses, unpredictable disease process and limited knowledge of long‐term consequences present significant challenges to parental mental health. We wanted to understand the emotional wellbeing of parents with children with CRMS/CFSPID to guide the mental health support offered within the service.

**Methods:**

Parents were invited to complete validated depression and anxiety screening questionnaires and answer an open‐ended question in writing or during a short interview. Qualitative responses were transcribed and analysed using thematic analysis.

**Results:**

Thirteen parents from nine families completed questionnaires and/or the interview. Two of the mothers had mildly raised scores on the questionnaires. Our interviews revealed five themes: difficulty adjusting to the label; concern about the future and its uncertainty; fluctuating states of anxiety; difficulty explaining the label; and satisfaction with the CRMS/CFSPID service.

**Conclusion:**

Our data reveal benign scores using objective screening tools, but the qualitative data paints a picture of potentially more significant impact on emotional wellbeing. We recommend screening parents from the time their child receives the label, and later the children themselves, for depression and anxiety and signposting to existing resources. Ultimately, a better understanding of the CRMS/CFSPID trajectory may enable us to better support families.

## 1 Introduction

Newborn screening (NBS) for Cystic Fibrosis (CF) has improved outcomes but introduces new diagnostic conundrums [1, 2]. The designation Cystic Fibrosis Transmembrane Conductance Regulator (CFTR) ‐related metabolic syndrome (CRMS), known in the UK and Europe as CF Screen Positive, Inconclusive Diagnosis (CFSPID) is used for infants with a positive NBS for whom follow‐up tests neither confirm, nor rule out, a CF diagnosis. In 2014 a global harmonised definition was reached [3]:

- An asymptomatic child with a positive CF newborn screen AND
- A sweat chloride < 30 mmol/L and two *CFTR* variants, at least one with unclear phenotypic consequences OR an intermediate sweat chloride (30–59 mmol/L) and one or zero CF causing variants.

Often following a positive NBS, older or subsequent siblings who do not have positive screening tests are tested‐ either on parental request, or suspicion of symptoms‐ and found to have the same *CFTR* variants as the infant designated CRMS/CFSPID. These siblings may be followed up in the CRMS/CFSPID service for consistency. A few children, born before the definition CRMS/CFSPID was agreed in 2014, were initially labelled as having CF and have subsequently, in some centres, been relabelled as CRMS/CFSPID [4].

CRMS/CFSPID is not a diagnosis but a designation or “holding label”. Most people with CRMS/CFSPID remain healthy, however, a minority will convert to a CF diagnosis or develop CFTR‐related disorders (CFTR‐RD) later in life (e.g. bronchiectasis) [5]. The uncertainty inherent in the designation makes it challenging to know how best to ‘medically oversee’ these children. The risk of over‐medicalising a healthy child must be balanced against that of missing a child who will reclassify as CF/CFTR‐RD [3]. Current recommendations include review at 6 months and then annual follow‐up of children with CFSPID then normally, at around 6 years, discharge to primary care with advice on when they should seek additional review [3], however we know from adult clinics that some people with the same combination of *CFTR* gene variants present later in life with symptoms and testing is consistent with a diagnosis of CFTR‐RD, or even CF [6, 7]. For this reason, our local practice is annual, remote contact via telephone or video through the later childhood years ahead of an in‐person review in early adolescence where conversations centre around ensuring that the young person is aware of the ‘holding label’ of CFSPID themselves, advice on the potential increased risks from smoking, how/when to seek further review or referral in the future, and potential impact on fertility in males. The guidelines also recommend offering a high‐resolution computed tomography (HRCT) scan at around five or 6 years of age [3], but, due to the radiation involved, this is a controversial recommendation given that many of these children are healthy. In our clinic we have access to multiple breath washout testing which is a useful alternative with no radiation [8], although HRCT is facilitated if parents wish.

In other diseases, diagnostic uncertainty has been shown to add significant mental health burden to parents [9, 10, 11, 12, 13]. The CRMS/CFSPID label usually comes at an already challenging time, caring for a young infant [12, 14] when rates of depression and anxiety symptoms are often higher in the general population [15]. In the UK, it is estimated that for every 300 infants with a positive CF NBS, 225 will have CF, 60 will be false positives and 15 will have a CFSPID designation. This number will rise significantly if the proposed introduction of gene sequencing detecting more *CFTR* mutations is adopted, and more families will likely be affected by the designation [16]. We wanted to better understand the emotional well‐being and experience of parents with children labelled as CRMS/CFSPID to improve the support we provide within our service.

### 1.1 Aims

- To assess the frequency of depression and anxiety symptoms in parents of a single‐centre CRMS/CFSPID cohort and to elicit parents' qualitative experiences of their CRMS/CFSPID journey to better understand how we can support their emotional wellbeing.

## 2 Methods

Parents were asked to complete the validated and widely used patient health questionnaire (PHQ‐8) and generalised anxiety disorder assessment (GAD‐7) screening tools, which are part of our standard of care for people with CF at our centre. Parents were also asked “Is there anything from the past regarding your CFSPID journey that you would like to share?”. The responses to this question were collected in writing, via telephone or face‐to‐face semi‐structured interviews. All responses were transcribed and coded in Excel. Thematic Analysis, as described by Braun and Clark, guided code and theme development of data from the open‐ended question [17, 18]. Analysis was conducted by two separate members of the team with experience of thematic analysis (LC, RD). If parents reported high scores in the screening questionnaires, or raised concerns in their open‐ended responses, appropriate referral pathways for support were followed. Every parent of a child meeting the 2014 agreed definition of CRMS/CFSPID at our centre was eligible and invited to participate. This project was conducted to understand the experience and thus needs of parents within our service and to determine the mental health screening and support we offer. As such, it was conceptualised as a quality improvement exercise and did not require formal ethical approval, however the purpose of the project was explained to all parents by someone experienced in consent procedures and they gave verbal consent to participate.

## 3 Results

Our centre cares for 14 children identified with CRMS/CFSPID through NBS as such, there were 14 eligible pairs of parents. In November and December 2023, 13 parents from nine families, nine mothers and four fathers, responded to the questionnaires, the open question, or both. Three of these families had another child identified to have the same genetic variants as their CRMS/CFSPID siblings, who are also followed up in our service for familial consistency. Participant identifiers, brief demographics and questionnaire scores are shown in Table 1.

**Table 1:** PHQ‐8 and GAD‐7 scores.

Participant Identifier (PID)	Relationship to child	Year child(ren) identified as having CRMS/CFSPID (mode of identification)	PHQ‐8 score	GAD‐7 score	Qualitative experience shared?
PID1	Mother	2019 (NBS) & 2019 (sibling, born 2019)	**8**	**7**	Yes
PID2	Father		4	2	Yes
PID3	Mother	2015 (NBS)	DNC	DNC	Yes
PID4	Mother	2019 (NBS)	1	3	Yes
PID5	Mother	2017 (NBS)	0	0	No
PID6	Mother	2018 (NBS) & 2018 (sibling, born 2014)	1	0	Yes
PID7	Father		0	0	Yes
PID8	Mother	2014 (NBS)	DNC	DNC	Yes
PID9	Father		0	0	Yes
PID10	Mother	2015 (NBS) & 2018 (sibling, born 2018)	4	**5**	Yes
PID11	Father		3	2	Yes
PID12	Mother	2016 (NBS)	DNC	DNC	Yes
PID13	Mother	2021 (NBS)	0	1	Yes

*Note:* PHQ‐8 severity scores: 0–4, none; 5–9, mild; 10–14, moderate; 15–19 moderately severe; 20–27 severe.

GAD‐7 severity scores: 0–5, none; 6–10, mild; 11–15, moderate; 16–21, severe

DNC Did not complete

One mother (PID 10) whose child was identified as having CFMS/CFSPID in 2015 on NBS and whose older child was found to have the same CFTR variants on parent requested genetic testing had raised scores on the GAD‐7. One mother (PID 6) with a baby identified as having CRMS/CFSPID in 2019 with a twin having the same variants reported raised scores on both tools.

Our data reveal benign scores in the objective PHQ‐9 and GAD‐7 screening tools, but the qualitative data paints a picture of a more complex impact on parental emotional wellbeing.

Although the raised scores were borderline, typically suggesting rescreen should be offered in a few months, the parents with raised scores were offered referral for psychological support. One was already receiving external support so declined, and one accepted the referral. Four parents were already receiving external psychological support, perhaps reflecting previous concerns with their emotional wellbeing. Additionally, four parents told us that their scores would have been higher in the first six to twelve months following NBS [12]. Our qualitative work identified five themes and four subthemes, summarised in Table 2.

1. Difficulty adjusting to the label.
  Following receiving the CRMS/CFSPID label, parents described the news as having an impact on their ability to enjoy their newborn baby, their marriages, their work, and their ability to connect with their social network.
  PID3: “We couldn't enjoy them as newborns”
  PID 7: “We sort of shut down. Didn't want to see anyone, didn't know what to tell anyone”
  Many reported still struggling to come to terms with the label and its possible implications.
2. Concern about the future and its uncertainty.
  Parents reported feelings of concern, fear and anxiety about the future particularly in relation to the uncertainty inherent in the CRMS/CFSPID label.
  PID8: “I think for CFSPID parents it is the unknown that is the biggest ongoing concern and worry”
  Parents reported relief that their child does not have CF, pressure to feel “grateful”, and conflicted emotions about ongoing uncertainty.
  PID1: “Getting the SPID diagnosis after expecting CF is like winning the lottery. But then you realise it's still serious”
  PID7: “There's difficulty admitting the strain of the CFSPID label as we're aware it's not so bad, so we shouldn't feel this way”
  PID11: “There is fear and living on a “knife edge”'. Its ok, but it might not be ok, and can change”
3. Fluctuating states of anxiety.

**Table 2:** Thematic analysis of the free response question.

Is there anything regarding your CFSPID journey that you would like to share?		
Theme	Subtheme	Example quotes
Difficulty adjusting to the label		PID2: It has impacted everything. Our marriage.
		PID3: We couldn't enjoy them as newborns
		PID 7: We sort of shut down. Didn't want to see anyone, didn't know what to tell anyone.
		PID11: I have definite psychological damage from first 8 weeks.
		PID11: My parents‐in‐law had to move in.
Concern about the future and its uncertainty		PID3: The limbo is exhausting
		PID8: I think for CFSPID parents it is the unknown that is the biggest ongoing concern and worry
		PID10: We weren't sure if this would be life threatening or mild or nothing
		PID11: There is fear and living on a “knife‐edge”. Its ok, but it might not be ok, and can change in heartbeat
		PID13: SPID is neither one thing or another, so it's tricky
Fluctuating states of anxiety	Linked to medical appts. and investigations	PID9: I didn't want to go to [the specialist hospital] because of what it represented
		PID11: Days at [the specialist hospital] take it out of me, I would need the day off the next day as I was so drained
		PID12: Sweat tests are always so stressful. What if this is the day?
	Linked to respiratory tract infections	PID1: We never know what to do if they get coughs, and we often panic
		PID3: We experience extreme fear and anxiety with a cough as we don't know what the implications are
		PID11: There's always what if “they start to get chest infections?”
Difficulty explaining the label	To non‐healthcare professionals	PID3: I have no resources to give, I have to educate people I encounter from what I have learned
		PID7: We would appreciate further support in explaining the diagnosis and reason for appointments to [our children]
		PID11: We would like guidance on how to discuss this with the children
	To healthcare professionals (HCPS)	PID1: With any health professionals apart from those at [the specialist hospital], we always have to explain
		PID11: We often have difficulty with other health professionals who are not trained to deal with CFSPID.
		PID12: Doctors and nurses are under the impression that he has CF and do not understand CFSPID, so he is treated as CF.
Satisfaction with the CFSPID service		PID6: [CNS and consultant] have been a great support throughout our journey
		PID8: We know who to speak to if we need to
		PID10: [Consultant and CNS] have put their arms around us many times literally and figuratively
		PID11: On the day we went to [the hospital], [CNS] greeted us out of the lift and made it all ok

Our data shows that, due to the uncertainty, families move in and out of states of fear and distress, which is, in itself, distressing. Particular times of distress and anxiety often related to medical tests and contacts with healthcare professionals (HCPs), which acted as concrete reminders their child risked developing serious illness.

PID1: “There's stress every time you have a sweat test or review. This one might show they have CF.”

Periods of respiratory infection, such as common colds, also led to increased anxiety.

PID3: We experience extreme fear and anxiety with a cough as we don't know what the implications are

4. Difficulty explaining the label.
*This was further divided into i) explaining CRMS/CFSPID to non‐HCPs for example school staff, family members, support networks and the child themself and ii) to HCPs without specialist CRMS/CFSPID knowledge.*
  PID3: “I have no resources to give, I have to educate people I encounter from what I have learned”
  PID11: “We often have difficulty with other health professionals who are not trained to deal with CFSPID”
5. Satisfaction with the CRMS/CFSPID service.

Parents universally reported satisfaction with the care they receive through the service. Many highlighted that this positive experience started before they had the results of the sweat test and the knowledge that their child fell into the CRMS/CFSPID category.

PID11: “On the day we went to [the specialist hospital], [the CNS] greeted us out of the lift and made it all ok”

Frequently praised aspects of care were specialist knowledge, accessible communication, and continuity of care.

## 4 Discussion

### 4.1 Objective Screening Tool Measures

Our group had benign scores on the commonly used PHQ‐8 and GAD‐7. Previous work in this area suggests that parents of children with CRMS/CFSPID have raised scores on depression and anxiety screening tools [19], with rates of depression and anxiety far closer to parents of children with CF than those of the general population. Parents in our cohort did state that these scores were likely to be higher in the early days following diagnosis, which suggests the newest CRMS/CFSPID guideline to screen parents at the first sweat test and at 6 months of age may pick up those parents in most need of support [20]. What is striking however, is the dichotomy between our cohort's benign results of well‐validated depression and anxiety measures, and the more complex feelings and experiences elicited through the interviews, which were consistent with previous qualitative work in this area [12, 19]. We do, therefore, add a note of caution that screening tools may miss some of the nuance of the emotional impact of the CFSPID designation and its attendant uncertainty.

### 4.2 Exploring the Five Qualitative Themes

Parents may experience loss, guilt, shock and anxiety when their infant has a positive newborn screening test or is diagnosed with a chronic illness [21, 22, 23], and these emotions are not linearly related to the severity of the illness [22, 24]. Parents, especially mothers, may experience high levels of emotional distress, sleep disturbance, depression and anxiety [14]. This impacts on relationships, emotional wellbeing and quality of life [21, 22, 24, 25]. Although CFPSPID is not an illness, and it is important we are clear in that message, parents experience similar stressors, and indeed the uncertainty and lack of control present additional challenges [12, 13]. A period of adjustment is needed, and parents need time to grieve what they have lost; their expectation of a healthy child.

It is important to identify where these feelings have become more pervasive or developed into adjustment disorder; an excessive reaction to stress that involve negative thoughts, strong emotions and changes in behaviour [26]. Incorporating mental health screening into the clinical assessment of parents may be a pragmatic way to identify those families who are experiencing significant impact on their emotional wellbeing and to identify those at risk of adjustment disorder. Reflecting on comments from parents, and consistent with current recommendations, this would be optimally initiated when parents come under the care of the CRMS/CFSPID service so they can identify those families who are struggling, and support parents through their potential grief and subsequent adjustment.

The CRMS/CFSPID designation is relatively new, therefore little is known about the long‐term natural history of the cohort. We know that a small percentage of individuals will develop CF/CFTR‐RD, but we currently do not know what this percentage is, and have no way to prospectively identify who is at risk [27]. The estimated risk of conversion seems to show wide variation in different cohorts even when based in a single country [4, 5, 27]. As such, the uncertainty experienced by parents is grounded in the reality of the situation.

Unpredictable disease process, limited knowledge of long‐term consequences, and prognostic uncertainty have been reported to increase perceived disease severity, lack of control, frustration and reduce optimism in other paediatric conditions [9, 10, 11, 13, 28]. Given our limited understanding of this cohort's future, providing education whilst being honest about our knowledge gaps may help to support families whilst providing a safe space to explore these existential issues. Acknowledging the potential impact on emotional wellbeing may also encourage people to share these more complex and challenging emotions.

Parents experience fluctuating states of anxiety, worse around investigations and appointments, highlights the need to ensure that the guidelines for medical management are proportionate, recognising the profound impact that overmedicalisation may have on parental, and later children's, mental health and wellbeing. Other times of anxiety and distress related to concern around the significance of coughs and respiratory tract infections. Healthy children can encounter multiple respiratory tract infections in the first years of life, especially if they, or siblings, attend nursery or school [29, 30]. However, for families living with CRMS/CFSPID, these infections can cause heightened anxiety both in terms of how they should be managed acutely, but also whether a simple infection signals the emergence of a more worrying diagnosis.

CRMS/CFSPID is a complex label. It does not fit society or HCP's common models of health and illness. This, combined with its rarity, means that it is often misunderstood and hard for parents to explain to those outside of specialist centre's teams. Suggestions from our cohort included awareness campaigns, especially for HCPs, and resources to use for schools, HCPs and with the child themselves to support discussions around the designation.

Although some public account bias must be considered, the almost universal satisfaction with the service demonstrates the benefit of managing these children and their families within specialist centres.

### 4.3 Strengths and Limitations

The use of mixed methods research is a strength of this study, as is its flexibility, which allowed participants to share their experiences in the way they found most comfortable. Our research team have strong grounding in CRMS/CFSPID and qualitative research, with contributions from across the multidisciplinary team allowing internal reflexivity. Our qualitative findings are plausible, often aligning with findings in population groups with similar characteristics or challenges. The PHQ‐8 and GAD‐7 are commonly used, validated tools, however, they are screening, not diagnostic tools. They also only give a brief snapshot into symptoms in the preceding 2 weeks, and our qualitative data suggests that there is significant variability over time. As with all qualitative research, we recognise a number of sources of bias in this study; researcher, social desirability and completion. We tried to minimise these by ensuring the interviewer was not known to the parents, and was not a part of the CRMS/CFSPID service and the analysis was conducted by two members of the research team. Furthermore, by necessity, our work involves small numbers due to a very small patient population.

## 5 Conclusions and Future Research

Although initially conducted as a systematic service improvement exercise, the findings are likely to be transferable to other CRMS/CFSPID populations across the UK and centres in other high resource countries and as such, we share our findings here. Our data do not show a high frequency of depression and anxiety in our cohort however, the qualitative data suggests further support may be required. Parents report a subjective experience of a significant effect on their emotional wellbeing, especially in the early years and around medical appointments and respiratory tract infection. As with much research in CRMS/CFSPID, it remains challenging to make blanket recommendations for the whole cohort. Any suggested input must balance the risk of overmedicalisation with the risk of missing opportunities to intervene; in this case by identifying those parents at risk of mental health morbidity secondary to the CRMS/CFSPID label. Routine screening using brief tools for depression and anxiety may be a pragmatic way to ensure longer term impacts on mental health and well‐being are captured, however due to our dichotomy between screening tool and qualitative findings we do add a note of caution with their use.

A longitudinal study, assessing depression and anxiety symptoms in parents after the positive NBS may be helpful to track these phenomena overtime. A matched healthy control and CF cohort could help to understand the mental health impact of the CRMS/CFSPID label in the early months versus the common mental health and emotional wellbeing challenges seen at this time, even in those with healthy babies. Our current work focusses on parental emotional wellbeing; it is also important to understand the impact on the children and adolescents themselves, and their siblings without the label. Another important avenue to explore is gaining a better understanding the natural history of CRMS/CFSPID, assessing whether sensitive biomarkers and outcome measures can predict risk. This knowledge would hopefully reduce some of the uncertainty that results in parental emotional distress. Our group are beginning work in this area (Cystic fibrosis: predicting long‐term health outcomes for children with an inconclusive diagnosis | Action Medical Research). As potential changes to the screening algorithms are made in several countries across the globe, more people are likely to receive the CRMS/CFSPID designation and thus the population numbers will increase [16]. Some people with the CRMS/CFSPID label are transitioning through to adult services, and it is important to know how best to follow them up and structure their care delivery.

Finally, several of our parents suggested that CRMS/CFSPID specific resources to help explain the label to would be helpful, including resources to support explaining CMRS/CFSPID to their children. The CF Trust and CF Foundation have excellent resources to signpost those newly identified as having CRMS/CFSPID (CFTR‐Related Metabolic Syndrome (CRMS) | Cystic Fibrosis Foundation and CFSPID resources hyperlinked). We flag and personalise these at our centre.

## Author Contributions

**Lynne Carty:** conceptualization, investigation, methodology, formal analysis, project administration, data curation, writing – original draft, writing – review and editing. **Rebecca Dobra:** writing – original draft, writing – review and editing, formal analysis, supervision. **Jackie Francis:** investigation, writing – original draft, writing – review and editing. **Michele Puckey:** conceptualization, writing – original draft, writing – review and editing, supervision. **Andy Bush:** writing – original draft, writing – review and editing, supervision. **Jane C. Davies:** supervision, writing – original draft, writing – review and editing.

## Conflicts of Interest

R.D. reports grants from UK Cystic Fibrosis Trust and Action Medical Research and honoraria for presentations from Vertex Pharmaceuticals. J.C.D. reports grants from UK Cystic Fibrosis Trust, Cystic Fibrosis Foundation, Cystic Fibrosis Ireland and EPSRC, payment or honoraria for lectures, presentations, manuscript writing or educational events from Vertex Pharmaceuticals, Boehringer Ingelheim, Eloxx, Algipharma, Abbvie, Arcturus, Enterprise Therapeutics, Recode, LifeArc, and Genentec. The other authors declare no conflicts of interest.

## Data Availability

The data that support the findings of this study are available from the corresponding author upon reasonable request.
